# Practice of dialysis access interventional nephrology procedures in the Asia-Pacific region: Getting lay of the land

**DOI:** 10.1111/nep.14236

**Published:** 2023-09-11

**Authors:** Sanjiv Jasuja, Maurizio Gallieni, Vivekanand Jha, Tushar Vachharajani, A. K. Bhalla, Jackson Tan, Cheih Suai Tan, Nabin Bahadur Basnet, Nalaka Herath, Ha Phan Hai An, Yong Soo Kim, Yaeni Kim, Krishnaswamy SampathKumar, Manisha Sahay, Raja Ramachandran, Suceena Alexander, Vinant Bhargava, J. Balasubramaniam, David Voss, Fredeick E. Ogbac, Atma Gunawan, Bak Leong Goh, Chih-Ching Lin, Jamshaid Khan, Ibrahim Shiham, Haroon Ayub, Min Aung Hein, Sarwar Iqbal, Nattachai Srisawat, Bihu Gao, Cherian Sajiv, Catherine Wilkinson, Thim Pichthida, D. S. Rana, Gaurav Sagar, Anupam Bahl, Samir Tawakley, Mragank Gaur

**Affiliations:** 1 Indraprastha Apollo Hospital, New Delhi, India; 2 DIBIC “L. Sacco”, Università degli Studi di Milano, Milano, Italy; 3 The Journal of VA, USA; 4 Fortis Kidney and Urology Institute, New Delhi, India; 5 John D. Dingell Veterans Affair Medical Center, Detroit, Michigan, USA; 6 Wayne State University, Detroit, Michigan, USA; 7 Sir Ganga Ram Hospital, New Delhi, Delhi, India; 8 Rimba Dialysis Center, Simpang, Brunei Darussalam; 9 Univeristy of Brunei Darussalam, Bandar Seri Begawan, Brunei; 10 RIPAS Hospital, Bandar Seri Begawan, Brunei; 11 Singapore General Hospital, Singapore, Singapore; 12 Nepal Mediciti Hospital, Karyabinayak, Nepal; 13 Teaching Hospital, Kurunegala, Sri Lanka; 14 Hanoi Medical University, Hanoi, Vietnam; 15 College of Medicine, St. Mary’s Hospital, Catholic University of Korea, Seoul, South Korea; 16 Meenakshi Mission Hospital and Research Centre, Madurai, India; 17 Osmania General Hospital, Hyderabad, India; 18 PGIMER, Chandigarh, India; 19 Christian Medical College (CMC), Vellore, India; 20 Kidney Care Centre, Tirunelveli, Tamil Nadu, India; 21 Middlemore Hospital, Auckland, New Zealand; 22 University of Brawijaya, Malang, Indonesia; 23 St Luke’s Medical Center, Quezon City, Philippines; 24 Dr Saiful Anwar General Hospital, Malang, Indonesia; 25 Hospital Serdang, Kajang, Malaysia; 26 Taipei Veterans General Hospital, Taipei, Taiwan; 27 Jamhuriat Teaching Hospital, Kabul, Afghanistan; 28 Indira Gandhi Memorial Hospital, Male, Maldives; 29 Hameed Latif Hospital, Lahore, Pakistan; 30 Defence Services Medical Academy (DSMA), Yangon, Myanmar; 31 BIRDEM General Hospital, Dhaka, Bangladesh; 32 Ibrahim Medical College, Dhaka, Bangladesh; 33 Chulalongkorn University, Bangkok, Thailand; 34 Affiliated Zhongshan Hospital of Dalian University, Dalian, China; 35 Alice Springs Hospital, The Gap, Northern Territory, Australia; 36 Cairns and Hinterland Hospital and Health Service, Cairns North, Queensland, Australia; 37 School of Medicine, International University, Phnom Penh, Cambodia; 38 Angkor Hospital for Children, Siem Reap, Cambodia

**Keywords:** Asia-Pacific, dialysis access, interventional nephrology (IN), survey, tunnelled-central catheter

## Abstract

**Aim:**

This cross-sectional survey aimed to determine the prevalence of Interventional Nephrology (IN) practice amongst nephrologists in the Asia-Pacific Region (APR), specifically related to dialysis access (DA).

**Methods:**

The Association of VA and intervenTionAl Renal physicians (AVATAR) Foundation from India conducted a multinational online survey amongst nephrologists from the Asia-Pacific to determine the practice of IN in the planning, creation, and management of dialysis access. The treatment modalities, manpower and equipment availability, monthly cost of treatment, specifics of dialysis access interventions, and challenges in the training and practice of IN by nephrologists were included in the survey.

**Results:**

Twenty-one countries from the APR participated in the survey. Nephrologists from 18 (85.7%) countries reported performing at least one of the basic dialysis access-related IN procedures, primarily the placement of non-tunnelled central catheters (n-TCC; 71.5%). Only 10 countries (47.6%) reported having an average of <4% of nephrologists performing any of the advanced IN access procedures, the most common being the placement of a peritoneal dialysis (PD) catheter (20%). Lack of formal training (57.14%), time (42.8%), incentive (38%), institutional support (38%), medico-legal protection (28.6%), and prohibitive cost (23.8%) were the main challenges to practice IN. The primary obstacles to implementing the IN training were a lack of funding and skilled personnel.

**Conclusion:**

The practice of dialysis access-related IN in APR is inadequate, mostly due to a lack of training, backup support, and economic constraints, whereas training in access-related IN is constrained by a lack of a skilled workforce and finances.

## 1 Introduction

Chronic kidney disease (CKD) is a complex and multifaceted disease, which if left untreated can lead to the development of kidney failure and cardiovascular disease.^1–3^ The majority of patients who develop kidney failure are treated by either haemodialysis or peritoneal dialysis.^1,4^ For effective haemodialysis, the patients require the creation of dialysis access (DA) such as arteriovenous fistula (AVF), AV graft (AVG), a tunnelled-central catheter (TCC) or non-TCC (n-TCC) that provides adequate blood flow through the procedure.^5,6^ For peritoneal dialysis, access is created by the placement of a peritoneal catheter. The creation and management of DA are vital interventional nephrology (IN) procedures^5^ that have been traditionally performed by trained specialists like vascular surgeons, cardiologists, urologists or interventional radiologists.^7^ In the past two decades, an increasing number of nephrologists, with appropriate training, have started to perform DA-related interventional procedures. Interventional nephrologists offer better access maintenance with minimized delays in the creation or correction with decreased access-related hospitalizations, limiting the use of temporary catheters, decreasing costs, and increasing patient convenience and longevity.^7–9^ There is a paucity of data regarding the practice of DA-related IN procedures amongst nephrologists, especially from the countries in the Asia-Pacific Region (APR). Our previous publications were limited to representative countries from South Asia (SA) and South-East Asia (SEA).^10–13^ Here, we report a multinational survey-based study, that collected data from countries from the APR concerning the available dialysis facilities, the ability of a nephrologist to create or treat dys-functional dialysis access and run training programs.

## 2 Materials and Methods

### 2.1 Data collection

Since the emphasis of our survey study was on DA-related interventions by nephrologists from the APR, a questionnaire (designed by the *A*ssociation of *VA* and interven*T*ion*A*l *R*enal physicians or AVATAR Foundation, India, www.AVATAR.net.in) focused on details of dialysis access interventions (distribution of treatment modalities, manpower and equipment availability, and monthly cost of treatment) and challenges in the training and practice of DA-related interventional nephrology (Supplementary Method) was circulated amongst:

- Council members of the Asia-Pacific Society of Dialysis Access (APSDA), in their individual capacity,
- For countries that were not represented by the APSDA council, interventional nephrologists recommended by respective national nephrology society members, and
- Willing to participate nephrologists from the Maldives, Cambodia, and Afghanistan (as there were no recognized national nephrology bodies or designated interventional nephrologists).

Responses received from 21 countries were provided based on the data available from national disease registries, local and regional studies, or an educated guesstimate of the problem. An attempt was made to capture multiple responses where the data source was not from national registries. Clarifications and additional input were sought through discussion and/or electronic communications. The collected data was compiled and statistically analysed. The absence of a response was categorized as ‘*Data not provided*’ or ‘*DNP*’ and censored from statistical analysis. Inconsistent responses received from different centres of an individual participating country were categorized as ‘*Variable*’ responses.

### 2.2 Statistical analysis

Countries in the region were grouped as low-income countries (LICs), lower-middle-income countries (LMICs), upper-middle-income (UMICs), and high-income (HICs) countries, as defined by World Bank, based on their Global Net Income (GNI) per capita status.^14^

For continuous data, the country-wise percentage was considered for comparison and presentation and the average was reported (for either the entire region or intergroup) as median [interquartile range or IQR]. Categorical data variables were presented as numbers and percentages keeping the entire region (21 countries) as the denominator.

## 3 Results

Nephrologists from 21 countries belonging to the Asia-Pacific geographical region participated in this questionnaire-based survey. An aggregate representation of the study responses is presented below.

### 3.1 Disease burden and practice patterns in the treatment of CKD

The average annual incidence rate of CKD-5 amongst the participating countries ranged between 75 and 523 pmp (Table 1). When compared, the LIC and LMICs reported an annual incidence rate for CKD-5 < 200 pmp (except India and Indonesia), whereas most of the UMICs and HICs—except China, Australia, and New Zealand—reported an annual CKD-5 incidence rate of >200 pmp. The highest incidence rate was reported from Taiwan (523 pmp) which is a HIC and the lowest from Afghanistan (75 pmp) which is a LIC (Table 1). Data were not available from Cambodia, Myanmar, Nepal, and Vietnam. Similarly, the average period prevalence rate of CKD-5 which ranged from 40 to 3587 pmp (Table 1) was much lower in LIC and LMICs as compared to UMI and HI countries.

Data was requested regarding the distribution of manpower and equipment availability with a monthly haemodialysis (HD) cost in participating countries. Overall, the availability of nephrologists as well as HD units was higher in HICs followed by UMICs, as compared to LMICs and the LIC. The number of nephrologists (pmp) in the participating countries ranged from as few as 34 in Afghanistan to as high as 71 in Taiwan. Afghanistan also reported the lowest number of HD units (0.37 HD units pmp) whereas the highest number (37.8 HD units pmp) was reported from Korea. The average monthly cost of HD ranged from 230 USD in Pakistan to 4868 USD in Australia (Table 1).

**Figure 1:**
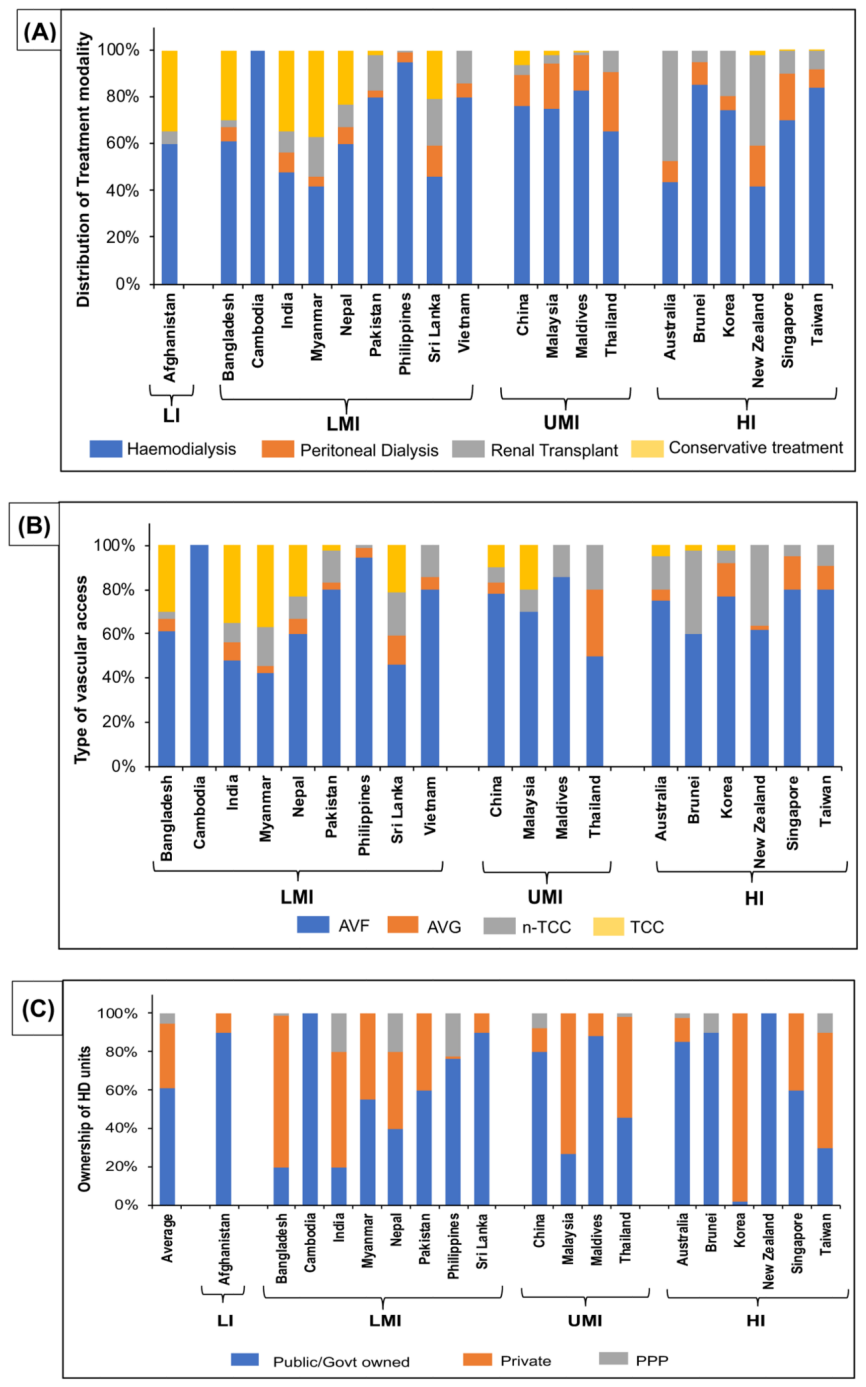
Basic information on the practice patterns in the treatment of CKD in participating countries. (A) Distribution of treatment modality amongst CKD patients (%), (B) type of access preferred amongst CKD patients (%), and (C) ownership of HD units, across participating countries from the Asia-Pacific region, based on their income status (HI, high income; LI, low income; LMI, low-middle income; UMI, upper-middle income). Countries that did not provide information were not represented.

Amongst the treatment modalities, HD was the most common treatment offered across APR irrespective of the income status. Peritoneal dialysis (PD) was preferred more in UMI and HI countries as compared to conservative treatments which were preferred in LIC and LMICs. Renal transplant as a treatment modality was highest in Australia, followed by New Zealand, and lowest in Cambodia and Maldives (Figure 1A). AVF was the most used access per unit (73%, per HD unit) followed by TCC (13%) in dialysis patients across APR, irrespective of the income status. While n-TCC was preferred more in LMICs, AVG was favoured in UMICs and HICs. High use of AVG as dialysis access was reported in Singapore, Korea, and Thailand (Figure 1B).

On average, most of the HD units across APR are public or government-owned (61 ± 30.84%), irrespective of the country’s income status. Countries where HD units were predominantly owned by the private sector included Korea (98%), Bangladesh (79%), Malaysia (73%), India (60%), and Taiwan (60%) (Figure 1C).

### 3.2 Current status of interventional nephrology

Dialysis access-related interventional nephrology or access IN procedures include special techniques, such as the insertion of catheters (TCC, n-TCC, and PD), the creation of AVF and AVG, and surveillance techniques for the maintenance of DA. We have categorized the access IN procedures as basic, which include TCC and n-TCC placement, and advanced procedures, which consist of endovascular interventions, creation of AVF and AVG, and placement of peritoneal catheters. According to our survey, 7/21 countries reported 100% of their nephrologist practicing either one or more of the basic access IN procedures; the Philippines reported the lowest (5%) (Table 2). The most common basic access IN procedure performed by nephrologists across all APR participating countries, irrespective of the country’s income status, was the placement of n-TCC placement (Median [IQR]: 75% [50–100]).

In contrast, the advanced access IN procedures were reportedly performed by a much smaller percentage of nephrologists. Amongst advanced access IN procedures, PD catheter placement was the most performed, especially in UMICs such as Malaysia, Maldives, and Thailand. Less than 10% of nephrologists from 7 countries were performing peripheral angiography and angioplasty, whereas <7.5% of nephrologists were conducting AVF surgery in 6 countries, and <5% from China, India, and Thailand were performing AVG surgery (Table 2).

Concerning equipment availability for performing dialysis and DA surveillance, most of the respondents (84.17%) reported having access to ultrasound machines, but only 30% of departments had access to Fluoroscopy/C-Arm or Cath lab (Table 2).

### 3.3 Access monitoring and event recording

Details about level 1 and level 2 access monitoring, personnel performing access monitoring, and the frequency of monitoring are presented in Table 3 and Supplementary Figure 1. Physical examination was the preferred method of first-level access monitoring by respondents from 19 countries. Only the respondents from 5 countries (23.8%), namely China, Pakistan, Singapore, Korea, and Taiwan, employed level 2 ECHO Doppler access monitoring on a routine basis and the procedure was performed by a small number of some nephrologists (<1%) only in China and Pakistan (Table 3 and Supplementary Table 1). In almost half of the responding countries (47.6%) the dialysis access monitoring was performed by dialysis nurses, mostly at random intervals (Supplementary Table 1).

### 3.4 Challenges in IN practice and future directions

Key challenges that hindered practicing IN amongst participating APR countries were lack of formal training (57.14%), lack of backup support (38%), time constraints (38%), lack of incentive (38%), and fear of medico-legal issues (28.6%) (Table 4 and Supplementary Table 2). Only 4 out of 21 countries (Myanmar, New Zealand, Singapore, and Sri Lanka) acknowledged positively that DA-related IN was a part of the general nephrology curriculum in their country. Mixed responses were received from Australia, Bangladesh, India, and Indonesia suggesting that DA-related IN training was available as part of the curriculum in some of the institutes but not others (Table 4 and Supplementary Table 2).

When questioned about the future of developing IN training hubs in current HD infrastructure and manpower in the country, 8 countries (38%) responded positively whereas 6 countries did not consider their HD infrastructure or manpower was ready to be used as training hubs. The biggest challenges in developing such training hubs were reported to be a lack of requisite manpower and finance (Table 4 and Supplementary Table 2).

## 4 Discussion

The present study provides a comprehensive report of the status and the gaps in the delivery of dialysis access-related interventional nephrology from 21 participating countries of the Asia-Pacific region (APR), collected mostly from nephrologists performing some access IN procedures in their respective countries.

According to the data provided by Global Burden of Disease Study,^3^ CKD is the third fastest-growing cause of death worldwide. Data from the current study documents an average incidence and prevalence rate of 231.0 [131.5–343] and 607 [327–1396] pmp, respectively, in the participating countries from the APR. The present numbers are similar to the incidence and prevalence of ESKD reported previously.^10^ Interestingly, we see a low prevalence and incidence rate in LMICs compared to UMI and HI countries, contrary to published literature,^15^ possibly due to late diagnosis and referral to nephrologists, and loss of patients due to poor follow-up (high attrition). It should be noted that most of the LMICs lack national/regional registries and data for current and past studies were accrued from previously conducted surveys which are themselves limited by various factors such as target population and method of data collection.

Although substantial variations in dialysis services across the world have been reported haemodialysis (HD) is the predominant form of dialysis preferred across the world whereas only 11% receive peritoneal dialysis (PD).^4,16,17^ Similar trends were observed when we surveyed the APR; more than 65% of the respondents reported HD as the preferred treatment modality. On the other hand, Thailand has initiated a ‘PD-first policy’ to cut down the dialysis expenses^18^; 25% of its patients use PD as a treatment modality. Interestingly, more than 20% of patients in countries from South Asia received conservative treatments possibly due to the lack of a Universal Healthcare program covering CKD resulting in financial burden on the patient, socio-economic conditions, difficulty in accessing a dialysis centre, especially in rural populations, or advanced disease stage at the time of presentation.

The scope of access-related interventional nephrology includes the use of special techniques for planning, creation, and maintenance of the dialysis access.^9^ According to our survey, AVF was reported to be the preferred DA (45%–92%). Singapore, however, reported AVG as a choice of dialysis access in 15% of patients. Although the updated Kidney Disease Outcomes Quality Initiative VA guidelines^19^ recommends the individualization of dialysis access (the right access for the right patient), AVFs are still considered superior to AVGs and catheters based on longer cumulative patency for dialysis and fewer frequency of interventions and infections.^20–22^ Another aspect of IN is the maintenance of VA; regular VA monitoring helps to extend and improve the life of the access.^20,23,24^ According to our survey, in 45.5% of the participating countries VA monitoring and/or surveillance were still conducted by dialysis nurses and not by consulting nephrologists. Even HI Countries such as Korea, and Australia, are known to use dialysis nurses exclusively.^25,26^

It is difficult to define the best interval of access monitoring; most short-term access surveillance studies failed to illustrate any benefit.^27–31^ We received variable responses from different institutes of the same country suggesting that in most participating countries the frequency of access monitoring was not defined; it is performed randomly, depending upon the patient’s condition. In most APR countries, Level 1 access monitoring was done, mainly by physical examination which is preferred as it is low cost and does not require additional equipment or dialysis personnel. In comparison, Level 2 ECHO-based Doppler access monitoring was used as a protocol in access monitoring by only 5 countries that participated in our survey. The ECHO-based Doppler access monitoring is a sensitive modality for HD access evaluation which is non-invasive, safe, inexpensive, and reproducible,^23,32^ but it requires more clinical skill, time, and equipment which could be a constraint for LIC and LMICs.

In our survey, all countries reported nephrologists practicing at least one basic IN procedure, but less than 50% of the participating countries responded as having nephrologists performing advanced IN procedures. The most common advanced procedure performed by nephrologists was PD catheter placement; nephrologists from 16/21 countries could perform it. In contrast, procedures such as creating AVF and AVG were being performed by a small number of nephrologists from China, India, Thailand, Nepal, and Bangladesh. Similarly, peripheral, or central stenosis management is the expertise of only less than 4% of nephrologists from Australia, China, Korea, Malaysia, India, Thailand, and Pakistan. The low frequency of nephrologists performing advanced IN procedures was probably due to the absence of IN subspecialty from the general nephrology curriculum. This was true for most countries; however, there was a disconnect in the case of New Zealand and Singapore where the frequency of nephrologists practicing IN was low despite the specialty being available in their training curriculum.

Amongst the challenges faced by trained interventional nephrologists, lack of backup support and time constraints were cited most frequently by the respondents. IN procedures are time-consuming and in countries where the number of nephrologists is low, the burden of clinical duties limits the time that can be dedicated for dialysis access care. This limitation can be overcome by increasing the number of trained nephrologists as well as technicians in a country, which could also compensate for the lack of backup support. It is necessary to have more training institutes or centres, and faculties to encourage more nephrologists to get trained in dialysis access-related IN procedures, especially advanced techniques.

Cost issues were another challenge faced by practicing IN reported mostly by LMICs. Dialysis care has often been correlated with a country’s GDP^17,33^; LICs and LMICs from SA and SEA are reported to have poorer dialysis infrastructure and inadequate manpower resulting in higher dialysis dropout rates.^12^

There were limitations to our study. Data from 10 out of 21 countries were collected from national registries whereas responses from the remaining countries were provided based on the data available from various published/non-published local or regional studies or an educated guesstimate. In addition, many countries could not provide data for some questions due to a lack of evidence.

Although limited by the number of participating countries, our study highlights a need for accelerating access to IN training programs, especially in advanced procedures. We believe that if a nephrologist trained in IN could perform access interventional procedures required by the patients himself; this would help secure the desired outcome, result in better patient care, minimize cost, and improve access patency and longevity.

## 5 Conclusions

Our survey identified significant heterogeneity in intervention nephrology practice patterns across the APR. Importantly only 8 countries had centres that could act as training hubs to provide IN training and the key challenges they are facing are the non-availability of both manpower and finance. An increment in government funding for developing IN training centres and collaboration with international professional organizations and industry partnerships will allow the countries of the APR, especially the middle to low and low-income, to train more nephrologists in doing procedures. Together, in collaboration with surgeons and radiologists, they will be able to build strong multidisciplinary teams dedicated to the well-being of the patients.

## Supplementary Material

Additional supporting information can be found online in the Supporting Information section at the end of this article.

Supplementary Material

## Figures and Tables

**Table 1:** Annual incidence rate, prevalence rate of CKD, and healthcare facilities available for treatment and management of CKD in APR.

Countries	Data from registry	Universal healthcare for CKD	Annual incidence rate for CKD-5 (pmp)	Prevalence rate for CKD-5 (pmp)	Number of nephrologists (pmp)	Number of HD units in the country (pmp)	Patient: technical manpower ratio in a HD unit	Average number of HD machines/ dialysis unit	Average monthly cost of HD (USD)
Low-income countries									
Afghanistan	No	No	75	75	0.34	0.37	7:1	6	DNP
Low middle-income countries									
Bangladesh	No	No	200	250	1.07	0.92	6:1	10	500
Cambodia	No	No	DNP	40	DNP	DNP	DNP	DNP	720
India	No	No	231	327	1.51	2.13	4:1	12	284
Indonesia	Yes	Yes	258	696	0.62	3.73	3:1	DNP	533
Myanmar	No	No	DNP	DNP	0.87	1.54	3:1	10	500
Nepal	No	Yes	DNP	DNP	2.15	1.99	4:1	8	240
Pakistan	No	Yes	200	450	0.87	0.65	4:1	8	230
Philippines	Yes	Yes	164	607	8.84	5.94	4:1	DNP	DNP
Sri Lanka	No	No	100	400	1.62	4.63	4:1	8	900
Vietnam	No	Yes	DNP	308	DNP	DNP	DNP	DNP	300
Median [IQR]			200.00 [148.00–237.75]	363.50 [264.50–567.75]	1.29 [0.87–2.02]	2.06 [1.08–4.41]	4:01 [4:1–6:1]	9.00 [8.00–10.50]	500.00 [262.00–626.50]
Upper middle-income countries									
China	Yes	Yes	114.6	659	4.14	4.14	7:1	35	DNP
Malaysia	No	Yes	245	1396	6.63	27.95	5:1	12	DNP
Maldives	No	Yes	260	950	6	30	6:1	8	700
Thailand	Yes	Yes	346	1342	DNP	9.61	DNP	DNP	800
Median [IQR]			252.50 [147.20–324.50]	1146.00 [731.75–1382.50]	6.00 [4.14–6.63]	18.78 [5.51–29.49]	6:1 [5:1–7:1]	12.00 [8.00–35.00]	750.00
High-income countries									
Australia	Yes	Yes	124	549	17.62	9.27	4:1	20	4868
Brunei	Yes	Yes	350	1800	15	17.5	5:1	20	2500
Korea	Yes	Yes	340	2006.4	55.38	37.81	DNP	29	1755
New Zealand	Yes	Yes	139	590	12.24	2.86	10:1	89	4700
Singapore	Yes	Yes	364	2030	16.95	33.9	4:1	10	2000
Taiwan	Yes	Yes	523	3587	71.15	31.44	4:1	30	1700
Median [IQR]			345.00 [135.25–403.75]	1903.20 [579.75–2419.25]	17.29 [14.31–59.32]	24.47 [7.67–34.88]	5:1 [4:1–10:1]	24.50 [17.50–44.75]	2250.00 [1741.25–4742.00]

Abbreviation: DNP, data not provided.

**Table 2:** Dialysis access-related interventional nephrology practice and equipment availability in participating APR countries.

	Nephrologist doing basic access IN procedures (%)			Nephrologist doing advance access IN procedures (%)							Nephrology department having access to following facilities (%)	
Countries	TCC placement	n-TCC placement		AVF	AVG	AVF/AVG salvage procedure	PD catheter placement	Central Venus stenosis management	Peripheral angiography & angioplasty		Ultrasound machine	Fluoroscopy | C-Arm
Low-income countries												
Afghanistan	0	50		DNP	DNP	DNP	DNP	DNP	DNP		DNP	DNP
Low middle-income countries												
Bangladesh	1	50		0.5	0	0.5	5	0	0		0	0
Cambodia	10	40		DNP	DNP	DNP	DNP	DNP	DNP		DNP	DNP
India	10	90		5	0.5	DNP	23	5	5		50	50
Indonesia	70	70		0	0	0	20	0	0		90	0
Myanmar	40	80		0	0	0	5	DNP	DNP		85	30
Nepal	3	90		1	0	1	4	DNP	DNP		80	40
Pakistan	10	100		0	0	0	5	1	1		100	5
Philippines	1	5		DNP	DNP	DNP	DNP	DNP	DNP		DNP	DNP
Sri Lanka	66	100		DNP	DNP	DNP	23	DNP	DNP		96	DNP
Vietnam	50	100		DNP	DNP	DNP	DNP	DNP	DNP		DNP	DNP
Upper middle-income countries												
China	25	30		7.5	5	5	DNP	3	4		90	90
Malaysia	20	75		DNP	DNP	DNP	90	15	10		50	50
Maldives	0	75		DNP	DNP	DNP	40	DNP	DNP		100	15
Thailand	20	100		1	1	1	30	1	1		100	1
High-income countries												
Australia	27.5	35		1	0	0	7.5	5	7.5			50
Brunei	20	100		0	0	0	20	DNP	DNP		100	0
Korea	20	62		0	0	0	20	7	9		100	100
New Zealand	22	50		0	0	0	11	0	0		90	35
Singapore	10	100		DNP	DNP	10	10	DNP	DNP		100	10
Taiwan	5	100		0	0	0	5	DNP	DNP		100	10

Abbreviations: AVF, AV fistula; AVG, AV graft; DNP, data not provided; n-TCC, non-tunnelled-central catheter; TCC, tunnelled-central catheter.

**Table 3:** First and second-level access monitoring and practice trends.

	**First level access monitoring (*n*, %)**						
		Yes	No		Variable	DNP	
	By physical examination	19 (90.5%)	0		0	2 (9.52%)	
	By QB stress test	1 (4.76%)	11 (52.4 %)		3 (14.3%)	6 (28.6%)	
	Dynamic venous pressure	5 (23.8%)	6 (28.6 %)		4 (19%)	6 (28.6%)	
	Static venous pressure	3 (14.3%)	8 (38%)		4 (19%)	6 (28.6%)	
	Kt/V online	10 (47.6%)	3 (14.3 %)		3 (14.3%)	5 (23.8%)	
	**Level 2 ECHO Doppler based access monitoring (*n*, %)**						
			**Yes**	**No**	**Variable**	**DNP**	
	ECHO Doppler monitoring of access performed as protocol		5 (23.8%)	10 (47.6%)	3 (14.3%)	4 (19%)	
	Is ECHO Doppler examination performed by nephrologists		2 (9.5%)	14 (66.7%)	2 (9.5%)	3 (14.3%)	

**Table 4:** Challenges to practice dialysis access-related IN and future directions.

	**Challenges in DA-related IN practice in participating countries (*n*, %)**				
		Yes	No	Variable	
	DA-related IN included in nephrology curriculum (*n* = 16)	4 (19.0%)	8 (38.0%)	4 (19.0%)	
	Time constraint (*n* = 17)	9 (42.8%)	7 (33.3%)	1 (4.76%)	
	Lack of backup support (*n* = 17)	8 (38.0%)	9 (42.8%)	-	
	Lack of formal training (*n* = 17)	12 (57.14%)	4 (19.0%)	1 (4.76%)	
	Cost issue^17^	5 (23.8%)	11 (52.3%)	1 (4.76%)	
	Fear of medical legal issues (*n* = 16)	6 (28.6%)	7 (33.3%)	2 (9.5%)	
	Lack of incentive (*n* = 15)	8 (38.0%)	6 (28.6%)	1 (4.76%)	
	**Future direction** (*n*, %)				
		Yes	No	Variable	
	Can the current dialysis unit be developed as a hub for training DA-related IN? (*n* = 16)	7 (33.3%)	7 (33.3%)	2 (9.5%)	
	What are challenges in developing new training hubs (*n* = 6)				
	Manpower	2 (9.5%)			
	Finance	-			
	Both	4 (19%)			

Abbreviation: DA, dialysis access.

## Data Availability

All data used to support the findings of this study are available from the corresponding author Dr. Sanjiv Jasuja at sanjivjasuja@yahoo.com upon request.

## Summary at a glance

A multinational survey of nephrologists from 21 countries in the Asia-Pacific region conducted to identify the current practice of dialysis access-related interventional nephrology (IN), competence to do basic and advanced access IN procedures, and challenges in the delivery and training of IN. Even though the majority of nephrologists could perform basic IN procedures, the Asia-Pacific region lacked proper training and practice of IN.
