# Supplementary material for: Practice of dialysis access interventional nephrology procedures in the Asia-Pacific region: Getting lay of the land
Source: Nephrology (Carlton). Author manuscript; Available in PMC 2024 Apr 18. (PMC7615839; doi:10.1111/nep.14236)
Supplement: Supplementary Material [file EMS195320-supplement-Supplementary_Material.zip › nep14236-sup-0001-methodsuppinfo.docx]

**Supplemental Methods**

Questionnaire for the multinational survey study conducted by the AVATAR foundation across countries from the Asia-Pacific region to assess the current status of dialysis access-related interventional nephrology (IN) procedures performed by nephrologists. The questionnaire is divided into 6 sections.

**Section A:DEMOGRAPHICS**

1. Email _____________________
2. Consent _____________________
   The information shared will confidential & clubbed with other institutions from your country to understand general trend.
   (To consent click Yes)
3. Name Of Country* _____________________
4. City _____________________
5. Name Of Institution _____________________
6. Type Of Institution _____________________
7. Institution Category _____________________
8. Name of participating Doctor _____________________
9. Mobile/Handset Number _____________________
   (Optional but requested)
10. Incidence rate CKD 5 | ESKD in your country _____________________
    (PMP = Per Million Population)
11. Prevalence rate of CKD 5 | ESKD in your country ____________________
    (PMP = Per Million Population)

##### Section B: BASIC INFORMATION

% Distribution of Renal Replacement Therapy (RRT) among CKD 5 patients in your country
(Total of all 4 columns should be 100%)

1. Hemodialysis % --------------------------------------------
2. Peritoneal Dialysis % --------------------------------------------
3. Transplant % --------------------------------------------
4. Conservative % --------------------------------------------
5. Total --------------------------------------------
6. Total number of nephrologists in the country(approximate) ________________
7. Number of hemodialysis units in the country --------------------------
8. Technical Manpower ratio to patients in Hemodialysis _________________ unit
9. Average number of hemodialysis machines per dialysis unit _____________
10. Tentative number of Hemodialysis machines in your country **(Optional) ---------**
11. The monthly cost of Hemodialysis in **(in USD $) --------------------------**
12. % Distribution of ownership of dialysis unit ---------------------------
13. Public | Government (%) ---------------------------
14. Private Sector (%) ----------------------------
15. Public-private partnership (PPP) (%) -----------------------------

**Section C: Dialysis Access-related Interventional Nephrology QUESTIONs**

1. Number of Prevalent Hemodialysis Patients in your dialysis program in the month of June 2022 ________________________________________

2. % Distribution of type of dialysis access if prevalent patients at your institute
(Total of all must be 100%)

AVF (%) ___________________________

AV Prosthesis | Graft (%) ___________________________

CVC | TCC (%) ___________________________

Acute Catheter (%) ___________________________

Any other (%) ___________________________

Total (%) ___________________________

3. In a country, what percent of nephrologists perform any dialysis access-related interventional nephrology procedure? ___________________________

4. Is dialysis access-related Interventional Nephrology (IN) part of the regular Nephrology training curriculum? ______________________

5.  The common challenges to the practice of access interventional nephrology?
You can choose more than one option

Time Constraint

Lack of Backup Support

No Formal Training

Cost Issues

Fear of Medico-Legal Issues

Lack of Incentive

Specialized for a Non-Interventional Nephrology Practice

Not Applicable to our Institution / Practice

6. What % of nephrologists in your country can perform following IN procedures?

Kidney Biopsy ___________________

Tunneled-central Catheter Placement ____________________

Non-tunneled central Catheter Placement ____________________

AV Fistula Surgery ____________________

AV Graft Surgery _____________________

AV Fistula / Graft salvage procedures(Surgical) _____________________

PD Catheter Placement _____________________

Central venous stenosis management in cath lab _____________________

Peripheral angioplasty & stenting _____________________

7.  What % of Nephrology training institutes have access to the following equipment?

- Ultrasound Machine _____________________
- Fluoroscopy / C- Arm / Cath lab _____________________

1. % of nephrologists performing cath-lab procedures in your country

- Central/Peripheral Venous Angioplasty with/without Stenting _________
- Renal Artery Angiography / Angioplasty / Stenting ____________________

**Section 4: FIRST LEVEL MONITORING of DIALYSIS access**

1. Who performs access monitoring -----------------------------------

2. How frequently access monitoring is being done? -----------------------------------

3. Do you perform Physical examination? -----------------------------------

4. Do you perform Qb stress test -----------------------------------

5. Do you measure dynamic venous pressures during a dialysis session?____________________________-

6. Do you measure static venous pressure before the start of dialysis? ---------

7. Do you measure Kt/V online    -----------------------------------

8.  Do you measure the time to hemostasis?  -----------------------------------

9.  Do you record out-of-vein punctures  -----------------------------

10.  Do you record intradialytic hypotension ----------------------------

11. Do you assess dry weight by clinical judgment or by BCM ------------------------

12.  Do you record thrombosis event -----------------------

**SECTION 5: SECOND LEVEL MONITORING OF DIALYSIS ACCESS**

1. Do you do level 2 ECHO doppler based access monitoring _________________

2.  Who performs ultrasound Doppler examination in the second-level access monitoring        ___________________________

3. What data is recorded in USG Doppler examination?

Venous Mapping & Diameter recording

Fistula Flow Rate

Brachial Artery Flow Rate

Any Other

4. What is the commonest abnormal finding you record out of following? _______________

5. How do you share critical and important information regarding dialysis access within the dialysis staff _________________________________

**SECTION 6: FUTURE DIRECTIONS**

1. Can your center be a hub for training other peripheral centers in performing dialysis access-related IN procedures? ___________________

If no, then please select reason(s):

Finance/funding issues

Lack of manpower/skilled personnel
