# Supplementary material for: Practice of dialysis access interventional nephrology procedures in the Asia-Pacific region: Getting lay of the land
Source: Nephrology (Carlton). Author manuscript; Available in PMC 2024 Apr 18. (PMC7615839; doi:10.1111/nep.14236)
Supplement: Supplementary Material [file EMS195320-supplement-Supplementary_Material.zip › nep14236-sup-0002-figures1.docx]

**Supplementary Figure 1. Access monitoring in participating APR countries.** *(A) Designated manpower for access monitoring and (B) Frequency of access monitoring.* (DNP-Data not provided)
