# Supplementary material for: Practice of dialysis access interventional nephrology procedures in the Asia-Pacific region: Getting lay of the land
Source: Nephrology (Carlton). Author manuscript; Available in PMC 2024 Apr 18. (PMC7615839; doi:10.1111/nep.14236)
Supplement: Supplementary Material [file EMS195320-supplement-Supplementary_Material.zip › nep14236-sup-0003-tables.docx]

**Supplementary Table 1: Dialysis access monitoring practice patterns amongst participating countries from the Asia-Pacific region, based on their income status.**

| **Countries** | **First Level Access Monitoring** | | | | | **Level 2 ECHO DOPPLER Based Access Monitoring** | | **Access Monitoring practice trends** | |
| --- | --- | --- | --- | --- | --- | --- | --- | --- | --- |
|  | **Physical Examination** | **QB Stress Test** | **Dynamic Venous Pressure** | **Static Venous Pressure** | **Kt/V** | **Does Your Institute Do Level 2 ECHO Doppler Monitoring Of Access Routinely** | **Is ECHO Doppler Examination By Nephrologists** | **Designated manpower for access monitoring** | **Frequency of access monitoring.** |
| ***Low Income Countries*** | | |  |  |  |  |  |  |  |
| Afghanistan | Yes | No | No | No | No | No | No | Dialysis Nurse | Every Dialysis Visit |
|  |  |  |  |  |  |  |  |  |  |
| ***Low Middle Income Countries*** | | | | | | | |  |  |
| Bangladesh | Yes | No | No | No | No | No | No | Dialysis Nurse | Every Dialysis Visit |
| Cambodia | DNP | DNP | DNP | DNP | DNP | DNP | DNP | DNP | DNP |
| India | Yes | DNP | Yes | DNP | DNP | Variable | Variable | Dialysis Nurse | Variable |
| Indonesia | Yes | No | Variable | Variable | Variable | No | No | Consultant Nephrologist | Variable |
| Myanmar | Yes | No | No | No | No | No | No | Variable | Variable |
| Nepal | Yes | NO | Variable | Variable | Yes | No | No | Variable | Variable |
| Pakistan | Yes | No | No | No | Yes | Yes | Yes | Dialysis Nurse | Randomly |
| Philippines | Yes | No | No | No | Yes | No | No | Dialysis Nurse | Every Dialysis Visit |
| Sri Lanka | YES | DNP | Yes | DNP | DNP | No | No | Variable | Variable |
| Vietnam | DNP | DNP | DNP | DNP | DNP | DNP | DNP | DNP | DNP |
|  |  |  |  |  |  |  |  |  |  |
| ***Upper Middle Income Countries*** | | | | | | | |  |  |
| China | Yes | Variable | Variable | Variable | Yes | Yes | Yes | Variable | Variable |
| Malaysia | Yes | Variable | DNP | DNP | Variable | DNP | DNP | Consultant Nephrologist | Variable |
| Maldives | Yes | DNP | DNP | Yes | Variable | Variable | No | Variable | Randomly |
| Thailand | Yes | Yes | DNP | DNP | Yes | No | No | Dialysis Nurse | 90 Days |
|  |  |  |  |  |  |  |  |  |  |
| ***High Income Countries*** | | | | | | | |  |  |
| Australia | Yes | Variable | Variable | Variable | Yes | Variable | Variable | Dialysis Nurse | Variable |
| Brunei | Yes | No | Yes | No | Yes | No | No | Dialysis Nurse | Randomly |
| Korea | Yes | No | No | Yes | Yes | Yes | No | Dialysis Nurse | 30 Days |
| New Zealand | Yes | No | Yes | No | Yes | No | No | Dialysis Nurse | 90 Days |
| Singapore | Yes | DNP | DNP | Yes | DNP | Yes | No | Dialysis Nurse | Randomly |
| Taiwan | Yes | No | Yes | No | Yes | Yes | No | Dialysis Nurse | 90 Days |

^DNP: Data not provided.^

**Supplementary Table 2: Challenges and future directions of dialysis access-related IN amongst participating countries from the Asia-Pacific region, based on their income status.**

| **Countries** | **Is DA-related IN Part Of The Training Curriculum In Your Country** | **Challenges In practicing access Interventional Nephrology** | | | | | | **Future Directions** | |
| --- | --- | --- | --- | --- | --- | --- | --- | --- | --- |
|  |  | **Time Constraint** | **Lack Of Backup Support** | **No Formal Training** | **Cost Issue** | **Fear Of Medical Lego Issues** | **Lack Of Incentive** | **Can the current center be developed as a hub for training in access IN?** | **Challenges To Develop Training Hub** |
| ***Low Income Countries*** | | | | | | | | | |
| Afghanistan | DNP | DNP | DNP | DNP | DNP | DNP | DNP | DNP | DNP |
|  |  |  |  |  |  |  |  |  |  |
| ***Low Middle Income Countries*** | | | | | | | | | |
| Bangladesh | Variable | DNP | DNP | DNP | DNP | DNP | DNP | No | Manpower |
| Cambodia | DNP | DNP | DNP | DNP | DNP | DNP | DNP | DNP | DNP |
| India | Variable | No | No | Yes | No | Variable | DNP | DNP | Manpower |
| Indonesia | Variable | Yes | No | No | Variable | Yes | Yes | Variable | DNP |
| Myanmar | Yes | Yes | Yes | Yes | Yes | No | Yes | No | Both |
| Nepal | NO | No | YES | YES | No | YES | No | No | Both |
| Pakistan | No | No | No | Yes | Yes | No | No | Yes | DNP |
| Philippines | No | No | Yes | Yes | Yes | Yes | No | Yes | DNP |
| Sri Lanka | YES | YES | No | YES | No | No | No | No | DNP |
| Vietnam | DNP | DNP | DNP | DNP | DNP | DNP | DNP | DNP | DNP |
|  |  |  |  |  |  |  |  |  |  |
| ***Upper Middle Income Countries*** | | | | | |  |  |  |  |
| China | DNP | Yes | Yes | Yes | Yes | DNP | Yes | Yes | DNP |
| Malaysia | No | Yes | No | No | No | No | Yes | DNP | DNP |
| Maldives | NO | YES | YES | YES | NO | No | No | No | Both |
| Thailand | No | Yes | No | Yes | No | Yes | No | Yes | DNP |
|  |  |  |  |  |  |  |  |  |  |
| ***High Income Countries*** | | | | | | | | | |
| Australia | Variable | Variable | No | Variable | No | Variable | Variable | Variable | DNP |
| Brunei | No | No | Yes | Yes | No | DNP/No | Yes | No | Both |
| Korea | No | No | Yes | Yes | No | Yes | DNP | Yes | DNP |
| New Zealand | Yes | No | No | Yes | No | No | Yes | No | DNP |
| Singapore | Yes | Yes | No | No | No | No | Yes | Yes | DNP |
| Taiwan | DNP | Yes | Yes | No | Yes | Yes | Yes | Yes | DNP |

^DNP: Data not provided.; DA: dialysis access^
